# From Metabolism to Medals: Contemporary Perspectives and Revisiting Carbohydrate Guidelines for Fueling Endurance Athletes during Exercise

**DOI:** 10.1016/j.tjnut.2026.101442

**Published:** 2026-02-25

**Authors:** James P Morton, J Marc Fell, Javier T Gonzalez, Mark A Hearris, Tim Podlogar, Jamie N Pugh, Gareth A Wallis

**Affiliations:** 1 Research Institute for Sport and Exercise Sciences (RISES) Liverpool John Moores University, Liverpool, United Kingdom; 2 Science in Sport, Blackburn, United Kingdom; 3 Centre for Nutrition, Exercise and Metabolism, University of Bath, Bath, United Kingdom; 4 Institute of Sport, Department of Sports and Exercise Science, Manchester Metropolitan University, Manchester, United Kingdom; 5 Department of Public Health and Sport Sciences, Medical School, University of Exeter, Exeter, United Kingdom; 6 School of Sport, Exercise and Rehabilitation Sciences, University of Birmingham, Birmingham, United Kingdom

**Keywords:** glucose, fructose, maltodextrin, cycling, marathon, ultraendurance

## Abstract

The effects of carbohydrate (CHO) intake on substrate metabolism, exercise capacity, and exercise performance have been studied for >100 y. From a metabolic perspective, the ergogenic effect of CHO intake is likely mediated by liver (and potentially muscle) glycogen sparing, maintenance of plasma glucose concentrations, and whole-body CHO oxidation rates, such that the required exercise intensity can be sustained for a longer duration thereby delaying fatigue. Accordingly, the 2016 sport nutrition guidelines from the American College of Sports Medicine recommend CHO intakes ≤90 g/h (from multiple-transportable CHOs, e.g., glucose/fructose mixtures), as targeted to exercise that is >2.5–3 h in duration. Although field observations report a trend for endurance athletes to consume (and experiment with) higher rates of CHO ingestion during training and racing (i.e., 120–200 g/h), the efficacy of such doses is not yet substantiated by current scientific research. Rather, contemporary research suggests that the upper limit of CHO intake could increase from 90 to 120 g/h (at least for trained participants), considering that both exogenous and whole-body rates of CHO oxidation can be increased with these higher ingestion rates. Such absolute doses may also modulate important physiological determinants of performance (e.g., durability and economy) across cycling, marathon running, and ultraendurance exercise. As such, the present paper provides a contemporary review of CHO metabolism during exercise, factors affecting exogenous CHO oxidation rates (i.e., CHO blend, ratio, format, environmental considerations, etc.) and sport-specific research (alongside personal author insights from practice), before presenting an updated and more nuanced model to guide CHO personalization strategies for endurance athletes. Directions for future research are also discussed, emphasizing the need for collaborative research to study both male and female athletes during ecologically valid exercise protocols that better address the real-world fueling challenges faced by elite athletes.

## Introduction

It is now an established practice for endurance athletes to consume carbohydrate (CHO) during exercise, especially in training and competitive scenarios where the exercise stimulus is prolonged in duration (i.e., >1 h duration) and of moderate-to-high intensity in nature [1]. Such “fueling” practices are firmly grounded in >100 y of scientific research, where the potential effect of CHO availability on “real-world” athletic performance was recognized as early as the 1920s with field observations from the seminal Boston marathon studies. For example, in the 1924 race, it was reported that a cohort of male runners presented with hypoglycemia on finishing the race and the researchers also reported “it is extremely significant that a correlation existed between the blood sugar level and the physical condition of the runner” (p. 1779) [2]. Importantly, hypoglycemia (and associated symptoms) was prevented in the 1925 race when the same males consumed additional CHO in the day before and during the race, with notable improvements in their physical condition and faster race time [3].

With the introduction of the muscle biopsy technique almost 40 y later, our understanding of CHO metabolism during exercise advanced considerably throughout the late 1960s [4,5], and 1970s [[6], [7], [8], [9], [10]]. Indeed, a series of studies from Scandinavia collectively demonstrated that a short-term high CHO diet increases muscle glycogen storage, elevated pre-exercise muscle glycogen availability enhances exercise capacity and performance, and that exercise depletes muscle glycogen in an intensity-dependent manner. Although the role of glycogen availability in modulating exercise performance was becoming increasingly accepted, it was not until the 1980s that research evaluating the effects of CHO ingestion “during” exercise regained momentum. In this decade, studies adopting prolonged cycling as the exercise modality demonstrated that the rationale to consume CHO during exercise (at least from a metabolic perspective) is largely based on the premise of preventing hypoglycemia and maintaining whole-body rates of CHO oxidation, thereby sustaining exercise intensity and delaying the onset of fatigue [[11], [12], [13], [14], [15]]. Additionally, the use of stable isotopes (e.g., [^13^C] and [6,6-^2^H_2_] glucose) throughout the 1980s [16,17], and 1990s [18] also allowed researchers to quantify the utilization of the differing CHO pools during exercise (e.g., liver, blood, and muscle), whereas the last 20 y of research transitioned toward evaluating the effects of different sugars/polymers (e.g., glucose, fructose, sucrose, maltodextrin, etc.) on exogenous rates of CHO oxidation [[19], [20], [21]]. Collectively, this accumulating body of research and developments in the field culminated in the most recent sport nutrition guidelines (published in 2016) recommending CHO intake during exercise at a rate of 30–90 g/h, with the dose and blend largely dependent on exercise duration [22]. It is noteworthy, however, these guidelines are almost a decade old and in building on previous research [19,20], more recent research [23,24] and anecdotal athlete reports [25] have suggested perceived benefits from even higher doses of CHO ingestion. In recognition of this apparent “fueling revolution,” the range of commercial product offerings now available to athletes has grown considerably, and it remains challenging for athletes, coaches, and practitioners to make evidence-based decisions in relation to optimally fueling for training and competition.

With this in mind, the aim of the present paper is to provide a contemporary review of CHO fueling during exercise. In taking a “Metabolism to Medals” approach, we first review the effects of CHO ingestion on substrate metabolism during exercise, before progressing to critically evaluate the factors that affect exogenous rates of CHO oxidation and the associated implications for exercise performance. We then share contemporary research and insights from applied practice (focusing on cycling, running, and ultraendurance) before presenting more nuanced CHO guidelines for athletes. Finally, we close by outlining directions for future research that will hopefully stimulate collaborative research worldwide to collectively address the “real-world” fueling challenges continually faced by elite athletes.

## CHO Availability and Substrate Metabolism during Exercise

### Effects of CHO feeding on whole-body substrate metabolism

Our understanding of substrate metabolism during exercise has been progressively advanced by the collective use of both invasive (e.g., muscle biopsies, isotope tracer infusions) and noninvasive techniques (e.g., indirect calorimetry, breath stable isotope enrichment, MRI, spectroscopy, etc.), the combination of which has allowed researchers to evaluate the utilization of specific substrate pools during differing exercise and nutrient scenarios. At the whole-body level, it is well documented that the predominant substrates for energy production during exercise are CHO and fat, where the contribution of each source toward whole-body energy utilization is largely determined by the interaction of exercise intensity [26], duration [27], training status [28], and substrate availability [29]. Indeed, such factors modify the utilization of both extramuscular (i.e., adipose tissue–derived free fatty acids, liver glycogen, and blood glucose) and intramuscular sources (i.e., muscle glycogen and intramuscular triglyceride), as mediated by hormonal control, cytokine-mediated signaling, and allosteric regulation of regulatory enzymes and proteins involved in substrate transport and metabolism.

**FIGURE 1:**
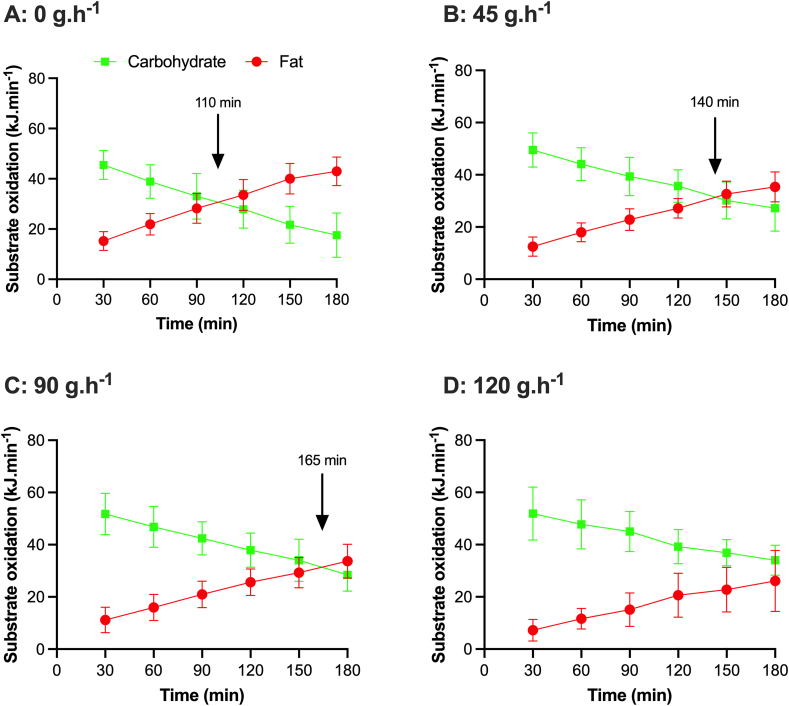
The effects of CHO ingestion during exercise on the contribution of CHO and fat to substrate metabolism. Data are redrawn and compiled from studies where male cyclists performed 3 h of submaximal exercise at 95% of lactate threshold and ingested (A) 0, (B) 45, (C) 90 or (D) 120 g/h. Note that ingestion rates of 45 and 90 g/h only *delays* the time to “crossover” compared with no CHO ingestion as indicated by the arrow on each panel. In contrast, 120 g/h *prevents* the crossover point, and CHO remains the predominant substrate. (A–C) Fell et al. [34]; (D) Hearris et al. [23]. CHO, carbohydrate.

In the context of evaluating the metabolic effects of CHO feeding “during” exercise, it is important to consider the nature of the exercise protocol and nutritional conditions of the chosen experimental design. This is especially relevant when considering that habitual macronutrient intake [30], pre-exercise muscle glycogen availability [31], and both timing [32] and composition of the pre-exercise meal [33] can all modify substrate metabolism in favor of both CHO and fat depending on substrate availability. In an attempt to mimic exercise conditions that are in accordance with nutritional guidelines and most likely applicable to the “competitive scenario” of the elite endurance athlete (i.e., 36 h of a high CHO diet and a high CHO pre-exercise meal), recent research from the first author’s laboratory evaluated the dose-dependent effects of CHO intake during exercise on whole-body substrate utilization (Figure 1). Such data demonstrate that CHO feeding [during 3 h prolonged steady-state cycling at 95% of lactate threshold (LT), in trained male cyclists] delays the “crossover point” during exercise i.e., the time-point during exercise whereby the predominant source of substrate metabolism shifts from CHO to fat. Moreover, within this given exercise context, consumption of 120 g/h *prevented* the occurrence of the crossover point during exercise [23], whereas 90 g/h only *delays* the time to crossover compared with 45 g and 0 g/h [34]. As such, it is apparent that CHO dependency during exercise is likely only maintained if CHO availability, itself, is also maintained, the result of which may have beneficial metabolic (i.e., economy, efficiency, durability) [35] and performance effects in that the target exercise intensity and desired race pace may be able to be maintained for a longer duration [14,36]. However, we acknowledge that a definitive CHO dose–response study and its effect on performance in training or competition-related scenarios is yet to be completed under standardized conditions in the *same* laboratory, as is the case for both male and female athletes. Nonetheless, in using a multisite trial, Smith et al. [37], reported a curvilinear dose–response relationship of CHO intake (0–120 g/h; 1:1:1 ratio of glucose, maltodextrin, fructose) on cycling performance (in a cohort of 51 recreationally trained cyclists and triathletes) with diminishing performance enhancement observed with intakes >78 g/h. In that study, CHO was ingested during 2 h of cycling at 95% of the workload corresponding to a blood lactate concentration of 4 mmol/L followed by a 20 km time trial. It remains to be proven whether such dose-response relationships are apparent in trained populations and during exercise scenarios that are more applicable to the intensities and durations associated with other endurance exercise scenarios.

### Effects of CHO feeding on liver glycogen utilization and blood glucose

The maintenance of high whole-body CHO oxidation rates associated with CHO ingestion is, in part, due to a maintenance of high circulating glucose concentrations. In the absence of CHO ingestion, prolonged moderate-to-high intensity exercise leads to the onset of hypoglycemia by ∼3 h [14]. The development of hypoglycemia in this context results from a mismatch between blood glucose rates of appearance (Ra) and disappearance (Rd), whereby glucose Ra is insufficient to meet the demands placed by glucose Rd. In the absence of CHO ingestion, liver glucose output is almost entirely responsible for glucose Ra, with contributions from hepatic glycogenolysis and gluconeogenesis [38]. As exercise duration continues, hepatic glycogenolysis depletes liver glycogen stores, which would reduce the relative and absolute contributions of hepatic glycogenolysis to glucose Ra. Because hepatic gluconeogenesis appears to show little absolute changes with exercise duration or intensity [38,39], hepatic glucose output (and consequently glucose Ra) can no longer meet the demand for Rd, and blood glucose concentrations will decline. For a 75 kg athlete exercising at ∼70% of peak oxygen uptake, hepatic glycogenolysis occurs at ∼30 g/h [38]. Because the liver has a maximal capacity to store ∼100 g glycogen [38,40], the onset of hypoglycemia at 3 h is consistent with the occurrence of hepatic glycogen depletion.

Accordingly, CHO ingestion during exercise has a profound effect on both delaying hypoglycemia and hepatic glycogen depletion. Indeed, as little as 20–30 g/h of glucose ingestion may begin to suppress endogenous glucose production and delay the onset of hypoglycemia [41,42]. The suppression of endogenous glucose production appears to be dose dependent with further suppression of endogenous glucose production up to ingestion rates of 90–120 g/h of glucose [41]. The suppression of endogenous glucose production suggests that there may be sparing of liver glycogen contents, which is supported by direct observations that ingesting large amounts of glucose (∼100 g/h) during exercise can completely prevent liver glycogen depletion [43]. Because glucose-fructose mixtures can further increase CHO availability during exercise (Composition of CHO blends section), it may be surprising that ingesting glucose-fructose mixtures at ∼100 g/h does not appear to further increase liver glycogen concentrations during exercise and instead is likely to be oxidized [43]. Indirect estimates have generally failed to detect differences liver glucose oxidation with increasing CHO ingestion rates or with differences in CHO type [44,45], although deduction of exogenous CHO oxidation from plasma glucose Ra suggests that glucose-fructose mixtures may reduce endogenous Ra during ultraendurance exercise from ∼0.6 to ∼0.4 g/min [46]. Therefore, it currently remains unclear what the dose response of CHO ingestion during exercise on liver glycogen sparing is, especially with CHO mixtures, and this information could further inform fueling strategies alongside other metabolic and performance data.

### Effects of CHO feeding on muscle glycogen utilization

Although CHO feeding has a clear effect on sparing liver glycogen utilization during exercise, effects on muscle glycogen sparing are smaller, more variable at the individual-study level, and appear to depend on exercise context (e.g., intensity, mode, and the muscle/fiber-type assessed). In running, muscle glycogen sparing has been consistently observed, with relatively modest CHO intakes (e.g., ∼60 g/h) reported to attenuate mixed and fiber-type specific glycogen depletion [47,48]. However, many individual studies employing prolonged cycling exercise, even with ingestion of large amounts (e.g., 100 g/h) of glucose-fructose mixtures, report no statistically significant reduction in mixed muscle glycogen utilization compared with placebo [14,34,43]. However, a recent meta-analysis of placebo-controlled crossover trials (31 studies; 48 unique effect sizes) indicates that CHO ingestion during prolonged endurance exercise produces a small but statistically significant muscle glycogen-sparing effect overall (SE difference ∼0.16), which translates to an estimated sparing of ∼24 mmol/kg dry wt over ∼100 min of exercise [49]. Notably, the direction of effect was generally consistent across studies, and no clear moderators (exercise mode, CHO type, ingestion rate, or pre-exercise glycogen) were identified. During lower intensity exercise (40% of maximal power output; ∼140 W) muscle glycogen concentrations can even be increased during cycling exercise when ingesting ∼80 g/h of sucrose [50], although this may not be apparent in untrained individuals [51]. Furthermore, there is indirect evidence that very high CHO ingestion rates may even stimulate skeletal muscle glycogen utilization, because tracer methods demonstrate that increasing glucose ingestion from 60 to 90 g/h may increase muscle glycogen oxidation rates during cycling [52]. Direct measures of muscle glycogen utilization with different doses and mixtures of CHO would provide additional support for this observation. The effects of CHO ingestion on muscle glycogen utilization therefore seem to depend on several factors. Muscle glycogen sparing is more commonly observed during running than cycling and with lower exercise intensities. Whether the goal is to spare or to stimulate muscle glycogen use will depend on the competition demands, since in the absence of reaching critically low muscle glycogen concentrations stimulating muscle glycogen utilization may provide greater use of a rapid and oxygen-efficient fuel. Furthermore, as exercise duration is prolonged and muscle glycogen decreases, there is an increasing reliance on blood glucose oxidation. Therefore, there may be a good rationale to optimize exogenous CHO availability under these scenarios to improve exercise performance during late stages of prolonged exercise.

### Effects of CHO feeding on adipose tissue lipolysis and intramuscular triglyceride utilization

The magnitude of the exercise-induced reduction in intramuscular triglyceride (IMTG) content (3 h prolonged steady-state cycling in trained male cyclists) in both type I and type IIA fibers is not affected by CHO consumption at rates of 45 or 90 g/h when compared with 0 g/h [34]. In contrast, CHO feeding suppresses circulating glycerol and nonesterified fatty acid (NEFA) concentrations in a dose-dependent manner, an effect likely mediated by suppression in hormone-sensitive lipase activity in adipose tissue [53], with decreases in lipolysis and increases in re-esterification. A dose-dependent suppression in circulating NEFA and whole-body lipid oxidation has also been reported across a smaller range of absolute CHO intake of 0, 20, 39, and 64 g/h, an effect also observed in trained male cyclists (during 2 h cycling at 95% of LT) [54]. In relation to running, data from the first author’s laboratory also reported that CHO intakes of 60, 90, and 120 g/h reduced circulating glycerol, NEFA and whole-body fat oxidation in male trained marathoners in a dose-dependent manner, as demonstrated during a 2 h treadmill running protocol where 90 min was completed close to race pace i.e., 94% of lactate turn-point [35]. When taken together, such data demonstrate that the reduction in whole-body fat oxidation associated with CHO feeding during exercise is most likely related to a reduction in rates of adipose tissue lipolysis [53], thereby reducing free fatty availability and/or reduced mitochondrial fat transport [55], as opposed to a reduction in IMTG utilization [56].

In summary, CHO ingestion during prolonged exercise exerts profound effects on the regulation of substrate metabolism, typically manifesting in maintenance (or higher rates) of whole-body CHO oxidation and an accompanying reduction in fat oxidation. Such alterations in substrate selection and utilization are likely a combination of sparing of endogenous CHO stores (notably liver glycogen), maintenance of plasma glucose concentrations and a suppression in adipose tissue lipolysis or plasma fatty acid oxidation. In this way, the rationale to consume CHO during exercise (especially across durations of 2–6 h) is largely based on the premise of sustaining the absolute rates of whole-body CHO oxidation that are necessary to maintain the desired exercise intensity for the required exercise duration.

## Factors Affecting Exogenous CHO Oxidation during Exercise

In the most recent sport nutrition guidelines, the upper limit of CHO intake is cited as 90 g/h (from products providing multiple-transportable CHOs), as recommended for those scenarios where exercise duration is >2.5 h [22]. Interestingly, earlier guidelines also suggest CHO ingestion rates between 40 and 110 g/h where exercise duration is >2 h [1]. In the latter case, the authors also advise glucose-fructose blends where CHO can be delivered in the form of “sports drinks/gels,” “solid sports food (low fiber and fat),” and/or “solid food.” Regardless of dose and format, however, it is noteworthy that CHO ingestion rates do not always translate to equivalent rates of exogenous CHO oxidation. As such, if an athlete intends to oxidize 90 g/h (i.e., 1.5 g/min), they should likely ingest CHO at rates of 90–120 g/h owing to the fact that oxidation efficiency is not uniform [23,24]. Accordingly, this section offers a contemporary review of the integrated factors that can affect absolute rates of exogenous CHO oxidation during exercise, all of which are key considerations when formulating CHO feeding strategies for athletes.

### Composition of CHO blends

Exogenous CHO oxidation rates with glucose or glucose polymer (maltodextrin) ingestion increase in a dose-dependent manner and plateau at peak rates ranging from 0.5 to 1.1 g/min when ingested at rates ≥1.0–1.2 g/min [52,57]. Dose-response studies have not been conducted with the other 2 dietary monosaccharides, fructose and galactose, but their oxidation in comparator studies feeding moderate CHO intakes (i.e., 0.65–1.2 g/min) is typically 25% and 50%–60% lower than that of glucose, respectively [[58], [59], [60]], likely due, in part, to the slower intestinal absorption of fructose [61], and/or the necessity for the conversion of fructose-to-glucose in the liver before oxidation [62,63]. Thus, at CHO intakes ≤1 g/min (60 g/h), single-source CHO (i.e., glucose or glucose polymer) is typically recommended. It should be acknowledged, however, *blends* of glucose and fructose (including as sucrose) or glucose and galactose (including as lactose) may be equally available as oxidizable substrates at such ingestion rates, especially during prolonged submaximal exercise in the moderate-intensity domain. For example, Hulston et al. [64], reported comparable rates of whole-body (∼2.1 g/min) and exogenous CHO oxidation (∼0.55 g/min) in trained male cyclists during 2.5 h of cycling (65% VO_2peak_) when ingesting 0.8 g/min of glucose or 0.54 g/min glucose plus 0.26 g/min fructose, respectively. As discussed previously [65], such data suggest glucose-fructose blends may also be applicable (or glucose polymer) even at these moderate ingestion rates due to the additional flexibility afforded if CHO intakes need to be increased as exercise progresses during a prolonged endurance event. It is noteworthy, however, consumption of a glucose and fructose blend (in gel format) at a rate of 60 g/h (40 g glucose + 20 g fructose) also reduced whole-body rates of CHO oxidation by ∼0.4 g/min during half-marathon running (1 h 50 min) when compared with 60 g/h of glucose only [66]. Although exogenous CHO oxidation was not quantified by these researchers, these data suggest that where there is the requirement to maintain high rates of whole-body CHO oxidation over shorter durations of higher exercise intensities (e.g., 1–2 h of exercise completed in the heavy and severe-intensity domains), it may be more advisable to focus on glucose and/or maltodextrin as the predominant source of CHO. Future studies are required to directly test this hypothesis.

Glucose-fructose blends offer clear advantages over glucose (or glucose polymer) when ingested at high ingestion rates (e.g., ≤90 g/h as per current recommendations; [22], because the 2 monosaccharides utilize distinct intestinal luminal transporters, thereby enhancing CHO absorption and systemic CHO availability during exercise [67]. Countless studies have demonstrated so-called multiple-transportable CHO blends to increase exogenous CHO oxidation during exercise by 20%–55% as compared with when an isoenergetic amount of CHO that uses a single intestinal transporter is ingested [68]. Part of the high total and exogenous CHO oxidation observed with multiple-transportable CHO ingestion can be attributed to fructose conversion to lactate, which is subsequently oxidized [69]; hence, the associated rise in blood lactate should not be misinterpreted to indicate a changing exercise intensity domain.

Some studies feeding multiple-transportable CHO blends at very high ingestion rates (i.e., 2.0–2.4 g/min, 120–144 g/h) have observed concomitantly high average peak exogenous CHO oxidation rates (1.60–1.75 g/min, 96–105 g/h) [19,23,24]. It is clear therefore that increasing CHO intakes above the currently recommended maxima of 90 g/h can substantially increase exogenous CHO oxidation during exercise. Also clear is that some athletes can self-select and tolerate consumption of CHO at doses >90 g/h in certain competitive situations. Whether very high CHO intakes (i.e., above 90 g/h) can further enhance performance beyond that possible within existing frameworks (i.e., 60–90 g/h for exercise lasting over 2.5 h duration) remains to be determined.

### Ratio of fructose-to-glucose

**FIGURE 2:**
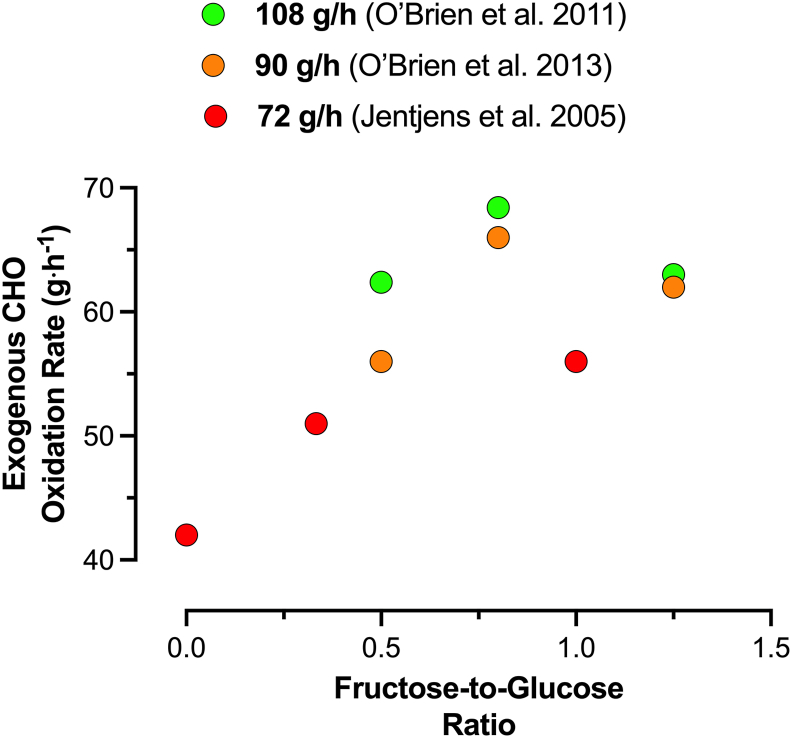
The effects of fructose-glucose ratio on rates of exogenous CHO oxidation during exercise. Data are redrawn and compiled from studies where male cyclists performed 2.5 h of submaximal exercise at 50% PPO [21,72], or 2 h at 57% PPO [73]. CHO, carbohydrate; PPO, peak power output.

Because glucose and fructose can interact with one another with respect to absorption and metabolism [70,71], the dose of each monosaccharide within a mixture may have an influence on exogenous CHO oxidation rates. To date, there have been few studies that have directly compared different ratios of glucose (polymers)-to-fructose on exogenous CHO oxidation rates and performance (Figure 2). Of the 3 studies that have directly compared differing ratios, there is a consistent pattern that increasing the fructose-to-glucose ratio from ∼0.4–0.5 to ∼0.6–1.0 results in a higher exogenous CHO oxidation rate across a range of total CHO intakes spanning ∼70–110 g/h [21,72,73]. Furthermore, the ratio that achieved the highest exogenous CHO oxidation also resulted in the greatest improvement in exercise performance [73], supporting the concept that increasing exogenous CHO availability and oxidation, whereas mitigating gut discomfort can enhance exercise performance. Furthermore, work is needed to understand whether there are other factors that dictate the optimal mix of CHOs to ingest during exercise, but at present, it seems that a ratio of fructose-to-glucose that is between 0.6 and 1.0 provides optimal exogenous CHO availability and oxidation.

### Format of CHO ingestion

Although the majority of CHO feeding studies have utilized fluids (i.e., drinks) as the format of ingestion, it is noteworthy that endurance athletes commonly consume CHO in a variety of forms, including drinks, semisolids (energy gels), and solids (energy bars, jelly chews) during both training [74] and competition [75]. In support of this approach, independent laboratories [23,76] have observed comparable exogenous CHO oxidation rates when ingesting fluids, gels, or chews (at rates of 108–120 g/h) during prolonged endurance cycling. Furthermore, ingesting a mixture of these formats (as reflective of the real-world fueling practices of endurance athletes) produces oxidation rates equivalent to each format in isolation [23]. Importantly, whether consumed in isolation or coingested together, these formats can achieve high rates of oxidation (>1.5 g/min) with minimal gastrointestinal (GI) distress, even at ingestion rates of ≤120 g/h. In contrast, lower exogenous CHO oxidation rates have been observed during the latter stages of prolonged endurance exercise when ingested in the form of a solid energy bar compared with a drink at ingestion rates of 93 g/h [77]. Solid CHO sources are also associated with increased subjective GI symptoms [77,78] and may be explained by delayed gastric emptying caused by the addition of protein, fat, and fiber within bar formats. Taken together, the reduced rates of oxidation and increased GI distress may contribute to the decline in performance observed with bar ingestion [78]. However, coingesting solid CHO sources with other formats such as drinks and gels appears to abolish any increased GI distress and performance decline observed with bar ingestion in isolation. As such, athletes are advised to adopt a mixed-format approach using a combination of fluids, gels, jelly chews and solids, providing them with the flexibility to select formats according to individual preference, overall CHO requirements, and race conditions or logistics. From a practical perspective, it should be recognized that CHO gels may be more prone to partial consumption (i.e., leftover product in the gel foil) and indirectly lead to lower ingestion rates [79], further supporting the recommendation for a mixed-format approach.

### Sex-specific considerations

There is increased appreciation that much of what we know about sports nutrition does not adequately address the potential for sex-specific considerations, with the area of CHO feeding during exercise no exception [80]. A limited number of studies have directly compared exogenous CHO oxidation during exercise in males and females. Two studies have not observed clear differences between males and females with respect to absolute peak exogenous CHO oxidation rates during exercise (i.e., <0.1 g/min lower in females and not statistically different from males; [81,82]. In contrast, 3 studies provide evidence of, or the potential for, higher absolute exogenous CHO oxidation rates in males compared with females, with males displaying ∼0.2 to 0.3 g/min higher rates [[83], [84], [85]]. The reason for contrasting results is likely to be multifactorial including study design features but perhaps most critically limited statistical power to draw firm conclusions. Indeed, all the studies compared a small number of each sex, typically between 6 and 8 males or females. Larger studies are needed to clarify if there are sex-differences in absolute exogenous CHO oxidation during exercise.

Alternatively, if we consider metabolic responses to CHO feeding in females per se, females exhibit increased exogenous glucose oxidation as ingested glucose dose is increased ≤60 g/h, with no further increase in oxidation with glucose ingestion at 90 g/h [52]. In 1 study, exogenous glucose oxidation was reported to not differ between females using oral triphasic hormonal contraceptives as compared with noncontraceptive users [85]. Furthermore, females appear able to achieve high oxidation rates (∼1.2 g/min, 72 g/h) with the ingestion of large amounts of multiple-transportable CHOs during exercise (2.2 g/min, 132 g/h) [84], though such doses when ingested in males are typically associated with larger rates of exogenous CHO oxidation and greater oxidation efficiency. That said, potential sex-differences aside, there does not appear to be any inherent limitation in the capacity to utilize ingested CHOs for energy during exercise, and indeed the ergogenic benefit of this practice is well-established [86]. Nonetheless, there is still a need to further investigate factors such as CHO dose and blend and effects of menstrual cycle and/or contraceptive use status on exogenous CHO oxidation in females. Additionally, considering the overall demand for CHOs in females in different contexts would ensure that female athletes can benefit from evidence-based research specific to their sex.

### Environmental considerations

Athletes are increasingly exposed to extreme environments such as heat and hypoxia both during training and competition. Both contribute to increased metabolic perturbations during exercise and can affect whole-body metabolism by raising relative exercise intensity at a given absolute power output thereby increasing the reliance on CHO as a fuel source [[87], [88], [89], [90], [91]]. At high altitude (i.e., >4000 m), the ability to oxidize exogenous CHOs is markedly reduced (i.e., by ∼20%–50%) at both relative and absolute exercise intensities [[92], [93], [94]]. Mechanistically, acute hypoxia suppresses both the systemic appearance of ingested CHOs and, more prominently, reduces whole-body glucose disposal/metabolic clearance during exercise, alongside higher circulating glucose and insulin concentrations [92]. Interestingly, this limitation appears reversible, as 3-wk altitude acclimation partially restores the capacity to oxidize ingested CHOs [93]. In practical terms, reductions in exogenous CHO oxidation rates are likely dependent on the magnitude of hypoxic stress, with existing studies typically conducted at high altitudes with large hypoxic stress that exceed those commonly experienced by most Olympic and endurance athletes both when training and racing. Furthermore, in many competitive contexts such as cycling or mountain running, exposure to altitude is transient, reducing the likelihood of meaningful metabolic impairment for the duration of the whole race. Therefore, although altitude acclimation remains advisable to optimize performance [95], there is no evidence that current CHO intake recommendations at altitude should differ from those applied at sea level.

Similarly, exercising in the heat at a fixed absolute workload can increase CHO demands through elevated glycogenolysis [[96], [97], [98]], an effect that is partially alleviated with heat acclimation [96,99]. Exogenous CHO oxidation rates, however, are consistently reduced during exercise in the heat [98,100] by 20%–30%, even when dehydration is prevented [101]. Despite reductions in exogenous CHO oxidation with heat stress, high CHO availability (i.e., before, during, and after exercise) remains important for sustaining performance in the heat, as supported by evidence that a higher-CHO diet during short-term heat acclimation improves subsequent time-trial performance in hot conditions [102]. The underlying mechanisms for reductions in exogenous CHO oxidation rates are presently not fully understood but may involve slowed gastric emptying, reduced intestinal absorption due to redistribution of blood flow away from the gut, increased muscle temperature leading to accelerated glycogen breakdown, and reduced muscle glucose uptake. From a practical perspective, it is important for athletes to acclimatize to the heat, as this confers numerous thermoregulatory and cardiovascular benefits [90], although evidence is lacking on the effects of heat acclimation on exogenous CHO oxidation. Although a reduction in exogenous CHO oxidation rates is consistently observed, the mechanisms responsible remain unclear; therefore, CHO intake recommendations should remain comparable with those used in temperate conditions. Nonetheless, a modest reduction in intake may be warranted in individuals experiencing GI discomfort which are more likely to occur in the heat, as discussed in the following section.

In contrast, evidence on substrate use during exercise in cold environments is mixed, with some studies reporting reduced CHO and increased fat oxidation [103], whereas others, particularly those combining cold and rain exposure, have demonstrated increased CHO utilization [104,105]. Despite these discrepancies in endogenous substrate use, exogenous CHO oxidation rates appear to be unaffected by cold exposure [106], with values comparable to those observed under thermoneutral conditions [84]. Consequently, current CHO intake recommendations should remain unchanged when exercising in the cold, especially considering that maintaining blood glucose availability is especially important in these conditions, as adequate CHO supply supports thermoregulatory heat production and helps prevent reductions in core temperature [107].

### GI considerations

The GI tract determines the availability of ingested CHO during exercise through the processes of gastric emptying, intestinal transport and absorption, and subsequent delivery to the portal circulation. Strenuous or prolonged exercise challenges these processes via splanchnic hypoperfusion and heightened sympathetic activation, compromising epithelial integrity, tight-junction regulation, motility, and absorptive capacity [108]. GI symptoms are reported by a high proportion of endurance athletes across a range of sports [[109], [110], [111]], with potential causes being multifactorial, including circulatory (ischemia and increased permeability), mechanical (repetitive impact), and nutritional factors including CHO dose, composition, and osmolality [112,113]. Understanding how these stressors interact with CHO feeding is therefore important to optimizing fueling strategies while minimizing GI compromise.

Exercise-induced compromise of intestinal integrity has been linked to the onset of GI symptoms [114], and in extreme cases, medical complications [115,116]. However, CHO ingestion during exercise has been shown to reduce markers of intestinal permeability compared with water ingestion, suggesting that exogenous substrate delivery may partially preserve epithelial integrity and limit barrier dysfunction under physiological stress [[117], [118], [119]]. Although moderate CHO ingestion may help preserve GI integrity, excessive or poorly tolerated intakes can have the negative effects of malabsorption and symptom development. When intestinal transport capacity [e.g., sodium-glucose cotransporter 1 (SGLT1) and glucose transporter 5 (GLUT5)] is exceeded, unabsorbed glucose/fructose can potentially raise small-intestinal water content and fermentation, provoking distension and GI symptoms [120]. Such GI symptoms linked to CHO intake are a potential cause for many athletes failing to meet the recommended intake during training and races.

A strategy to mitigate exercise-associated GI symptoms and improve tolerance to high CHO intakes is the structured and repetitive exposure of the gut to CHO feedings, often referred to as “gut-training.” Across the 8 interventions included in the most recent systematic review [121], research protocols involving CHO ingestion during exercise (30–90 g/h) consistently reduced gut discomfort. These adaptations occurred without measurable changes in gastric emptying rate [122] or epithelial injury markers [123]. Although there has so far been no evidence for increases in exogenous CHO oxidation, lowered indices of CHO malabsorption (assessed by postexercize breath hydrogen) [124,125] indicate that improvements may reflect enhanced transporter activity (e.g., SGLT1, GLUT5) and reduced sensory feedback from luminal CHO accumulation. Such adaptations are also likely reflective of daily dietary intakes as increases in exogenous CHO oxidation during exercise have been shown in athletes following a high CHO diet (8.5 g/kg/d) compared with a control group (5 g/kg/d) [126]. Practically, progressive exposure to higher-CHO intakes during training and high dietary CHO intakes in the days and weeks before competition likely improves tolerance and reduces the incidence and severity of GI symptoms.

### CHO personalization

Current CHO intake guidelines adopt a “one-size fits all” dosing approach, as it has long been assumed that interindividual variation in exogenous CHO oxidation is insignificant and cannot be explained by factors such as body size or metabolic rate [68]. More recently, however, substantial interindividual variability in the capacity to oxidize ingested CHO during exercise was reported [23,127,128]. A recent study directly tested the relationship between body size and exogenous glucose oxidation by comparing smaller (<70 kg) and larger (>70 kg) trained cyclists ingesting 90 g/h of ^13^C-labeled glucose during prolonged exercise [127]. Larger athletes oxidized substantially more exogenous glucose (mean ∼45 compared with ∼33 g/h; mean difference ∼13 g/h) and measures of body size (mass, height, calculated body surface area) correlated strongly with peak exogenous oxidation, indicating body size is indeed a determinant factor of exogenous glucose oxidation, although it does not explain all the interindividual variability. Importantly, when larger athletes reduced intensity to match the absolute workload of smaller athletes, the difference slightly attenuated but did not disappear, implying that both body size and absolute power can contribute to the observed effect, as had been speculated previously [129].

Podlogar et al. [128] recently tested whether direct measurement of an individual’s peak exogenous glucose oxidation could be used to prescribe a personalized glucose dose that delivers comparable oxidation while reducing total intake. In their proof-of-concept protocol, endurance-trained participants completed 2 X 150-min steady state cycling trials at ∼95% of LT1. In the first trial, participants consumed a high glucose dose (90 g/h) enriched with ^13^C to determine each participant’s peak exogenous glucose oxidation rates. This value was then used to calculate a personalized glucose dose based on an assumed oxidation efficiency (∼80%), which was given in the second trial. The mean personalized glucose dose was 65 ± 10 g/h (∼28% <90 g/h) yet produced indistinguishable peak exogenous glucose oxidation rates compared with the high dose. The protocol required prolonged exercise (i.e., 2.5 h) at a sustainable steady-state intensity so that exogenous glucose oxidation rates could rise and reach a peak, which only occurs after ∼2 h of exercise [23,24,57,128,130].

This personalization work focused only on the glucose-based component of CHO ingestion, typically provided as glucose polymers such as maltodextrin, representing just 1 component of most sports nutrition products, which usually contain both glucose- and fructose-derived CHOs. Because glucose and fructose use different intestinal transporters, an athlete’s peak oxidation rate for 1 CHO type (e.g., glucose) may not predict their peak for the other (e.g., fructose). Although a 1:0.8 glucose-fructose ratio appears to approximate the average combined oxidation capacity of both substrates, interindividual variation likely exists in the relative contribution of each, meaning that some athletes may benefit from slightly different proportions. Moreover, ^13^C colabeling of both monosaccharides in a single test would not allow separation of their respective oxidation rates, making glucose-only testing the most pragmatic first step. In principle, ^14^C tracers could be used to distinguish both substrates simultaneously [73], but because these are radioactive, their use in human exercise studies is not ethically or practically acceptable.

In applied practice, personalization offers a promising approach to maximize exogenous CHO availability without exceeding an individual’s absorptive or oxidative capacity and thereby reducing the risk of GI discomfort or unnecessary stimulation of endogenous glycogen use [20,45,52]. Although current work demonstrates the feasibility of personalized dosing for glucose and its polymers, further research is required to understand individual variability in glucose-fructose utilization and to determine whether similar approaches can be applied to shorter-duration exercise, where exogenous oxidation may not reach a plateau. Some athletes may be capable of tolerating and oxidizing higher-CHO doses than present guidelines suggest (e.g., >120 g/h), but identifying such individuals requires an individualized approach.

## Contemporary Insights from Sport-Specific Research and Applied Practice

The practical application of CHO feeding strategies for endurance athletes should recognize that specific endurance sports have different physiological and metabolic demands as well as practical and logistical constraints associated with CHO consumption during exercise e.g., access, format, and frequency of opportunity to feed during exercise. Accordingly, this section presents a review of contemporary research findings aligned to cycling, marathon running, and ultraendurance exercise but also shares some personal reflections, insights, and observations from our work as practitioners across these sports. It is noteworthy that while we are witnessing a trend for athletes to consume (and experiment with) higher rates of CHO ingestion during training and racing (i.e., 120–200 g/h), it is also acknowledged that the efficacy of such practices is not yet substantiated by current scientific research.

### Cycling

The ergogenic effect of CHO availability on exercise capacity [14,34], and performance during cycling [36,37], has long been recognized. In recent years, such research has also progressed to evaluating the effects of CHO intake on sport-specific physiological determinants of performance such as durability or fatigue resistance [131]. In this context, durability can be defined as the resilience to the deterioration of physiological variables and performance capability (i.e., mean and peak power output for a given duration) during or after prolonged exercise [131]. Interestingly, Clark et al. [132] reported that CHO consumption at a rate of 60 g/h (albeit in nonelite endurance participants) prevented the decline in critical power (CP) that occurred after 2 h of cycling within the heavy intensity domain, an effect apparent in the absence of muscle glycogen sparing. In elite and professional road cyclists performing 4 h of intermittent cycling with 100 g/h CHO, Ørtenblad et al. [133], reported ∼10% and ∼6% reductions in 6-min time-trial mean power and sprint peak power, respectively. Overall, fat and CHO oxidation rates during the ride were not strongly related to these decrements, although higher fat oxidation in the fourth hour was modestly associated with a larger decline in 6-min power. In addition, Spragg et al. [134] reported that in male professional cyclists, better durability (smaller reductions in CP after a severe-intensity fatiguing protocol with ∼60 g/h CHO) was associated with lower CHO oxidation at 200 W as well as higher gross efficiency, $\dot{V}$O_2max_ and threshold power outputs. Such data collectively suggest that an increased capacity for fat oxidation during moderate-intensity exercise (so as to spare glycogen utilization and maintain CHO availability later in exercise) may contribute to improved durability during prolonged exercise models. Such patterns of substrate utilization are, of course, characteristic of the metabolic adaptations associated with endurance training and indeed, provide further rationale for the application of CHO periodization during training [135]. In such instances, the aim is to stimulate metabolic adaptations that subsequently promote fat oxidation during the moderate and heavy intensity domains while retaining the capacity for high rates of CHO oxidation during the transition from the heavy to severe domain, the latter achieved through increased CHO availability late in exercise but also the training-induced adaptations in enzymatic and mitochondrial capacity to facilitate high rates of CHO oxidation when required.

**FIGURE 3:**
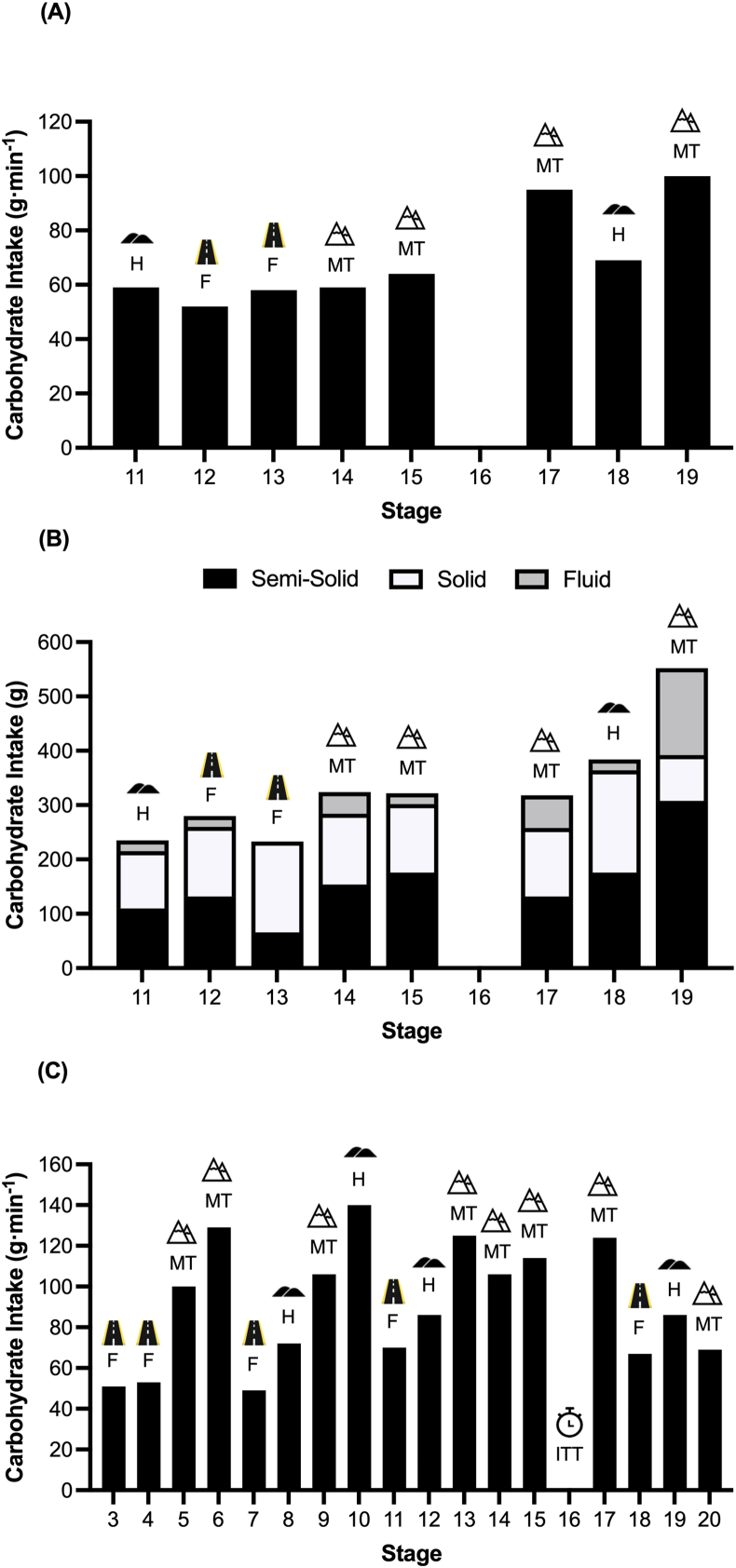
CHO intake of the 2018 Giro d’Italia winner Chris Froome during stage 11–19 expressed in (A) g/h and (B) absolute quantity of CHO consumed from semisolids (i.e., gels), solids (i.e., bars and rice cakes) and fluids (Morton, personal communication). (C) CHO intake of a World Tour cyclist during stages 3–20 of the 2023 Tour de France (Fell, personal communication). CHO, carbohydrate; F, flat stage; H, hilly stage; ITT, individual time-trial stage; MT, mountain stage.

In recognition of the ergogenic effects of CHO, the habitual intakes of professional cyclists have often been reported as 60–90 g/h, as is the case for both Grand Tours [[136], [137], [138]] and shorter stage races [75,139]. Notably, these investigations typically report mean values that are averaged across riders and stages, thereby overlooking the potential for intra-race variation and the likely periodization of CHO intake across stages of differing physiological demand. Indeed, in accordance with the differing stage profiles (i.e., flat, hilly, mountain, summit finish, etc.) and associated variations in day-to-day stage duration (i.e., 4–7 h) and energy expenditure (which can vary between 3000 and 6000 kJ/d) [[140], [141], [142]], contemporary approaches often involve the deliberate manipulation of both daily CHO intake and “on-bike” CHO intake, with the goal of simultaneously optimizing fueling and maintaining the desired body mass for key stages. Such nutritional periodization formed the basis of Chris Froome’s 2018 Giro d’Italia victory (Figure 3A and B), whereby CHO intake varied from 52 to 96 g/h, the latter occurring on the most demanding stage of the race (stage 19; on-bike energy expenditure of 6180 kJ). The public disclosure of detailed Grand Tour fueling case studies has occurred alongside a clear increase in-race intensity, with recent editions of the Tour de France and other World Tour events being among the fastest in history, both in overall average speed and in record-fast stages. This combination has likely encouraged practitioners to experiment with higher-CHO intakes in the modern peloton (Figure 3C). Furthermore, recent reports indicate that a stage winner on a 2025 Tour de France stage consumed ∼116 g/h CHO, and some riders are now reportedly training their GI system to tolerate 200–220 g/h [25]. This apparent divergence between practice and the current evidence base likely reflects *1*) the pursuit of marginal gains in an increasingly competitive sport and *2*) the extreme energetic demands of multihour stages, where higher-CHO intakes may help riders better match intake to expenditure and support daily energy balance [143], even if this does not necessarily confer an immediate performance benefit. As outlined in CHO personalization section, substantial interindividual variability exists in exogenous CHO oxidation capacity; notably, recent tracer work [127], reported an individual oxidizing ∼90 g/h of ingested glucose, implying that, when combined with fructose, some athletes may have the capacity to oxidize amounts exceeding 120 g/h. It is therefore plausible that a subset of riders can absorb and oxidize CHO at rates above conventional recommendations, providing a rationale for personalized approaches to CHO intake among athletes seeking optimal CHO delivery.

Although the practical implementation of such high CHO intakes is facilitated by advances in sports nutrition products [23], the physiological implications for CHO absorption, oxidation, GI comfort and performance remain to be fully characterized. On the basis of the dosages studied to date, a CHO intake of 90–120 g/h currently represents the most evidence-based recommendation for the upper limit of CHO intakes for the majority of athletes [23,24], but the anecdotal practices described above and the growing focus on personalized CHO intake suggest that these guidelines will likely evolve. Moreover, because race-deciding moves often now occur earlier (e.g., with 20–50 km remaining rather than within the final 5–10 km), the requirement to sustain high power output and maintain CHO dependency is probably greater than ever. Future research should therefore employ more ecologically valid exercise protocols [144], and nutritional interventions (e.g., integrating durability assessments and evaluating higher-CHO doses and formats) to refine our understanding of how CHO availability influences metabolism and performance in cycling. Additional areas of practical relevance for practitioners to consider are outlined in Table 1 [79,145,146].

**TABLE 1:** Emerging practitioner-relevant nutrition considerations and future research needs for prescribing and assessing CHO intake of cyclists in stage and race settings

Practical nutrition consideration or area of future research	Context	Scientific reference
Is it plausible that muscle glycogen synthesis occurs during races or stages?	Owing to the variable intensity of road cycling, riders may spend extended periods at low power in the peloton or freewheeling on long descents. During these phases, CHO intake may support some degree of glycogen resynthesis, as reduced muscular demand allows a portion of ingested CHO to be directed toward glycogen synthesis rather than immediate oxidation.	Kuipers et al. [50] Yaspelkis et al. [145]
Implications for estimating CHO intake during races or stages	In-race CHO intake is often estimated from riders’ self-reported consumption of prepackaged sports foods and drinks. This approach may overestimate actual CHO intake by ignoring residual product in wrappers, partially consumed gels and unfinished bottles.	Lanpir et al. [79]
Does CHO feeding pattern influence metabolic and physiological responses during races or stages?	Practitioners often calculate average hourly CHO intake by dividing total CHO consumed by stage duration. This assumes a constant intake rate, which rarely reflects the reality of racing. Attacks, weather, and tactical moments can create substantial hour-to-hour variation in CHO intake. The physiological consequences of these fluctuations, and of different ingestion strategies (e.g., large infrequent boluses, regular moderate feedings, or continuous small “drip” feeding) remain unclear.	Jones et al. [146]

Abbreviation: CHO, carbohydrate.

### Marathon running

In contrast to cyclists, the habitual CHO intakes of marathoners [75,147] during competition are considerably less (e.g., reported mean intakes of ∼35 g/h) and at the elite level, the predominant form of CHO delivery is fluids. Such comparably low CHO intakes may be driven by a combination of behavioral factors such as lack of knowledge of CHO guidelines [148], and challenges associated with the physical opportunity to consume CHO during the race itself, but also an increased prevalence of GI symptoms associated with running [149]. Nonetheless, in considering the challenge of running the “2 h” marathon, recent modeling data have suggested the current ≤90 g/h recommendations would be insufficient for 65% of the modeled runners [150].

**FIGURE 4:**
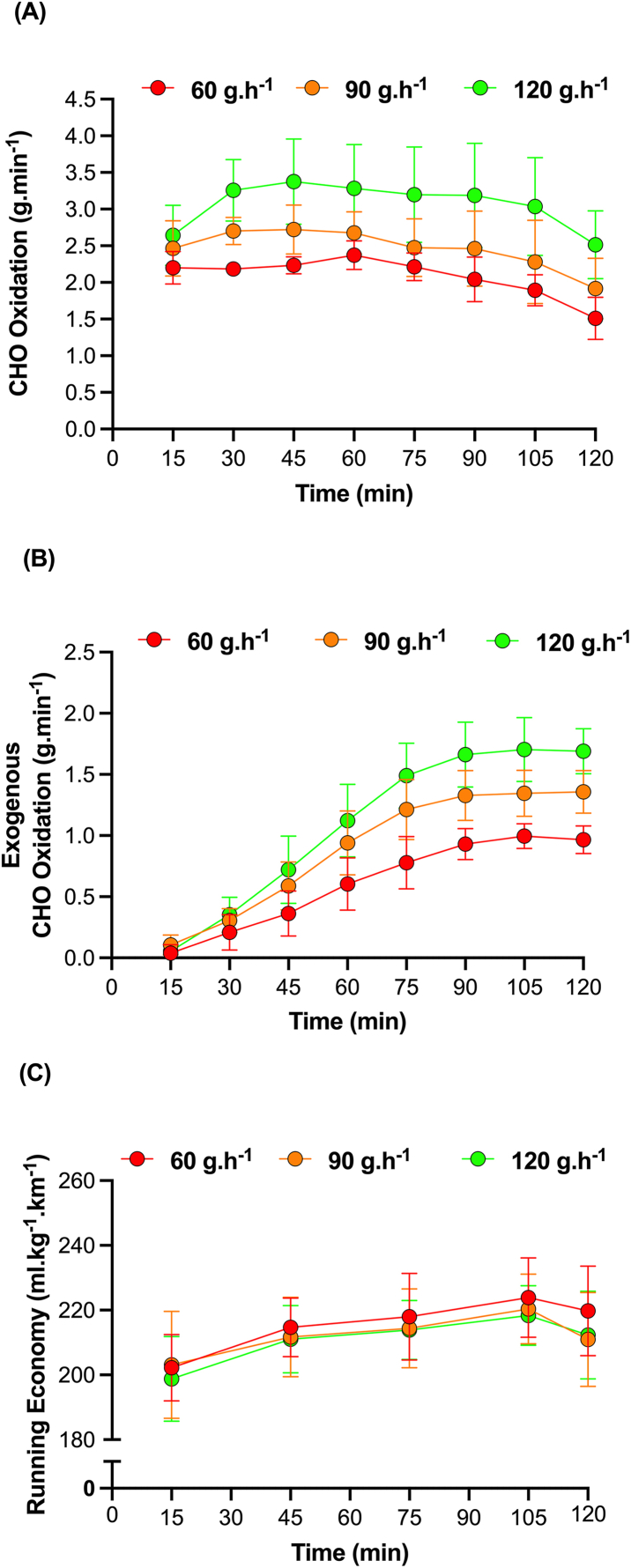
The effects of CHO ingestion (at rates of 60, 90, and 120 g/h) during running on (A) whole-body rates of CHO oxidation, (B) exogenous CHO oxidation, and (C) running economy. Data are redrawn from Ravikanti et al. [35] and are representative of trained male runners completing 2 h of treadmill running (an initial 15-min period at 95% of LT, a subsequent 90-min period at 94% of LTP and a final 15-min period at 95% of LT). In the second hour of exercise, the contribution of CHO toward total energy expenditure was significantly different between conditions such that CHO accounted for 65%, 51% and 43% in the 120, 90, and 60 g/h trials, respectively. CHO, carbohydrate; LT, lactate threshold; LTP, lactate turn-point.

Indeed, although both the half-marathon [151] and marathon [152] distances are considered as CHO-dependent events, recent data from the first author’s laboratory has demonstrated that CHO dependency is only maintained with higher ingestion rates of 120 g/h [35]. In this regard, Ravikanti et al. [35] evaluated whole-body CHO and fat oxidation, exogenous CHO oxidation, and running economy in a cohort of male elite marathoners (all marathon PB faster than 2 h 30 min) while ingesting 60 (maltodextrin only), 90 (2:1 maltodextrin-fructose) or 120 g/h (1:1 maltodextrin-fructose). The authors reported a dose-dependent effect of CHO intake on whole-body and exogenous CHO oxidation and a 3% improvement in running economy with 120 g/h compared with the 60 g/h trial (Figure 4). Nonetheless, moderate symptoms of GI discomfort were reported with all doses of CHO, and peak symptoms for nausea, stomach fullness, and abdominal cramps were greatest in the 120 g/h trial. When taken together, such data demonstrate a metabolic advantage of CHO consumption at doses of 90–120 g/h, though the effects of such higher ingestion rates on marathon performance (in both males and females) remain to be determined, especially in consideration of the associated GI symptoms.

From a practical perspective, the pattern and frequency of CHO intake during the marathon often occurs at set distance intervals (e.g., every 5 km, typically corresponding to 15 min intervals) where each athlete will have their personalized fueling options available for collection. Accordingly, the attainment of the chosen fueling targets in-race is likely dependent on a personalized fueling plan that has been trialed and tested in prior races and at comparable intensities and durations in training. It is noteworthy, however, there is already a trend for marathoners to ingest higher absolute CHO doses during racing (i.e., > 90 g/h, usually from concentrated drinks), undoubtedly due to both developments in research but also insights from elite athlete practice e.g., Eluid Kipchoge publicly disclosed racing with 60–100 g/h for the Nike Breaking 2 and the INEOS 159 project. Nonetheless, further research is required to evaluate the optimal dose, CHO ratio, frequency and format of CHO delivery (i.e., fluids compared with gels) that is ergogenic for male and female runners.

### Ultraendurance and multisport events

Ultraendurance and multisport events place substantial metabolic and GI demands on the athlete, but they can differ markedly in the constraints they impose on fueling. Ultraendurance events (e.g., mountain and road ultramarathons, multiday races) can involve extreme durations and limited access to food and fluids, whereas multisport events (e.g., triathlon/duathlon) span a wide range of race durations (from Olympic-distance to long-course) and introduce discipline-specific feeding restrictions (e.g., no intake during the swim, variable feasibility during transitions and running). Collectively, these factors create large interevent and intraevent variation in CHO intake opportunities and tolerance, and therefore in the amount and pattern of CHO that can be consumed during competition [153,154]. These constraints also determine whether athletes rely primarily on “collected” fuels (e.g., frequent aid-station access) or must “carry” substantial CHO supplies (as is common in mountain ultramarathons and multiday events), thereby influencing both the quantity and consistency of CHO intake.

Evidence informing nutrition strategies in multisport events remains limited, and current guidelines are largely extrapolated from single-sport data and applied practice. Triathlon presents additional discipline-specific constraints on fueling. During the swim, feeding is typically not feasible; therefore, prerace CHO availability and early intake on the bike may be particularly important for whole-race CHO provision. The cycling leg generally provides the best opportunity to deliver high CHO and fluid intakes, whereas GI symptoms are common in long-course events and may become more problematic during the run [155,156]. This occurs despite evidence that exogenous CHO oxidation rates are similar between prolonged running and cycling at comparable relative intensities [157], suggesting that discipline-specific differences in realized intake during triathlon events are driven primarily by practical constraints on ingestion while running and GI tolerance (rather than oxidation capacity per se). In athletes prone to GI symptoms, a pragmatic strategy may be to front-load CHO intake on the bike and modestly reduce intake late in the bike and/or early in the run to minimize symptom risk, although this approach is speculative and should be individualized. In long-course triathlon, the run is initiated after several hours of preceding exercise, and accumulated physiological strain (e.g., progressive dehydration and thermoregulatory/cardiovascular stress) may further exacerbate GI discomfort; dehydration has also been shown to impair gastric emptying and increase GI complaints during running [158]. Moreover, heat stress and dehydration can influence CHO metabolism during prolonged exercise (e.g., whole-body CHO oxidation and glycogen use), although direct mechanistic evidence in multisport settings remains limited and warrants further investigation [159].

Although the relative exercise intensity of ultraendurance exercise is lower than that of shorter endurance races [[160], [161], [162]], the absolute CHO cost associated with the metabolic demands of such prolonged durations has not been well characterized, particularly in elite athletes. Nonetheless, case-study observations have provided some insight to the CHO requirements to compete in such events. For example, mean whole-body CHO oxidation in a well-trained male athlete during the first 64.5 km of a 160 km mountain trail ultramarathon was ∼2.1 g/min, equivalent to 68% total energy expenditure [163]. In a world champion male 100 km runner, exercise intensity was estimated to be 74% volume of oxygen maximum (VO_2max)_ for ∼6.5 h, with a mean whole-body CHO oxidation of 1.6–2.3 g/min, providing 68%–83% of total energy expenditure throughout the race [164]. In a simulated long-duration triathlon, mean whole-body CHO oxidation in a male world-class triathlete was 4.1 g/min during 4 h of cycling, but reduced to 2.3 g/min during 135 min of subsequent running with ∼55% of total energy being derived from fat oxidation [165]. Sustaining whole-body CHO oxidation rates in this case study would be possible with maximal pre-exercise muscle glycogen and very high CHO consumption during exercise (144 g/h) although it has been suggested that the absolute exercise intensity could also be sustained from fat oxidation [166]. Interestingly, the maximal rate of fat oxidation has also been shown to be an independent predictor of ultraendurance performance [167], and evaluation of substrate metabolism in such elite case-studies provides further evidence that the ability for high rates of fat oxidation during prolonged exercise is a necessity to maintain the required rates of energy production.

In consideration of the extreme metabolic demands, there is an obvious requirement for CHO consumption during ultraendurance exercise. Indeed, field studies report that higher rates of CHO consumption (66 ± 27 g/h) are associated with a greater likelihood of race completion compared with nonfinishers (42 ± 23 g/h) [168,169], as well as improved physiological and ergogenic effects across a range of ultraendurance events e.g., more stable blood glucose and faster finishing speeds in a 100-mile ultramarathon [170], and greater total distance covered during 24-h running [171]. However, when the hourly CHO intakes are considered, many of these studies report values below the recommended intakes of 90 g/h of multiple-transportable CHOs. Furthermore, for races with mean finish times ∼12–24 h, intakes of between 30 and 66 g/h have been reported [168,[172], [173], [174]]. Such low intakes are likely due to a number of factors including the range of athletes studied (many are recreational, nonelite), race logistics, athlete knowledge, GI symptoms, and an apparent aversion toward sweet-tasting energy gels and sports drinks with longer durations due to changes in taste preferences [[175], [176], [177]]. However, there are also case-studies of elite athletes consuming 80–100 g/h in both 100-mile [164], [24]-h [178] competitions. An average CHO intake of 96 g/h has also been reported by a world-class male athlete completing a 5-d 960 km race [179].

In contrast to cycling and marathon running, the effects of CHO ingestion rates >90 g/h on physiological responses, substrate metabolism, and exercise performance are not well studied in either the laboratory or field settings. Nonetheless, it is noteworthy that CHO ingestion rates of 120 g/h in male elite runners during a mountain marathon reduced postrace markers of exercise-induced muscle damage (EIMD) compared with 60 and 90 g/h [180]. Given that EIMD has been suggested to be a major determinant in ultraendurance performance [180], this may be another mechanism for improved performance with higher-CHO doses.

From a practical perspective, a critical success factor underpinning ultraendurance performance depends on the ability to sustain CHO intake while also minimizing GI symptoms under diverse and unpredictable race conditions. Indeed, the practical delivery of CHO is multifaceted in such a wide array of events and may change athlete-to-athlete, race-to-race, and within sections of races. Evidence from laboratory, field, and elite case-studies suggests that well-trained athletes who progressively train the gut could tolerate ≥90 g/h of multiple-transportable CHO, implemented through personalized and adaptable fueling plans. Indeed, there are recent anecdotal reports of athletes consuming >120 g/h in some of the most difficult 100-mile races exposed to high environmental temperatures and altitudes [181], although empirical research (in both males and females) is needed to support the efficacy of such strategies. Our reflections from supporting such athletes are that the most common determinants of whether high CHO intakes are achieved are, in fact, an initial *belief* in the importance of CHO for promoting performance, as well as race fueling preparation, gut tolerance and logistics associated with the race itself. In addition, access to a well-coordinated crew support, innovations in delivery (such as cold or semifrozen CHO drinks used strategically in hot environments) and the availability of well formulated and more palatable sports nutrition products can also promote in-race fueling behaviors. Further research is required to explore the barriers and enablers to fueling in both training and racing scenarios.

## Toward Contemporary CHO Guidelines for Athletes

**FIGURE 5:**
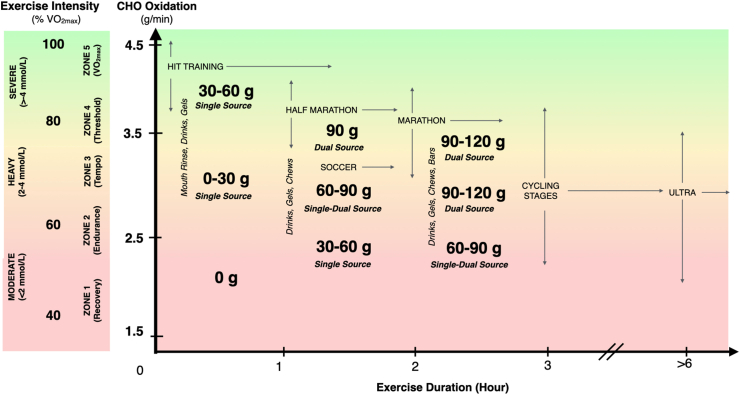
Theoretical model to guide CHO feeding strategies for endurance athletes. This model considers the absolute quantity (i.e., g/h) and format of CHO ingestion (i.e., drinks, gels, chews, bars) that may be best suited to maintaining the whole-body rates of CHO oxidation that may sustain the target exercise intensity (and economy/efficiency/durability, etc.) for the required exercise duration and thereby optimize performance. Exercise intensity is depicted according to 3 common approaches such as exercise intensity domains, % of VO_2max_ and endurance training zones, where upward and downward arrows are indicative of the range of intensities and rates of CHO oxidation associated with each sporting scenario. Similarly, exercise duration is segmented to hourly zones where rightward arrows are indicative of the range of durations associated with each sporting scenario. It is noted that the absolute rates of whole-body CHO oxidation and the range of associated exercise intensities and exercise durations are representative of published data from male athletes that span the training status of tier 2 (trained/developmental), 3 (highly trained/national), 4 (elite/international level), and 5 (world class) athletes [182]. Accordingly, absolute rates of CHO oxidation are likely not applicable for female athletes and further research is required to address this gap (Summary and Directions for Future Research section). CHO, carbohydrate; VO_2max,_ volume of oxygen maximum.

The 2016 guidelines from the American College of Sports Medicine recommend CHO intakes of 30–60 g/h (for endurance exercise and “stop and start” sports lasting 1–2.5 h) and 90 g/h, the latter dose targeted to “ultraendurance” exercise that is >2.5 to 3 h in duration. However, in view of the recent research developments discussed within the present review, it would now seem timely to provide updated and more nuanced guidelines that also consider the interaction between the target exercise intensity and typical durations associated with specific endurance and ultraendurance sporting scenarios (Figure 5) [182]. In this context, we define ultraendurance exercise as durations >6 h, where exercise scenarios spanning 1–6 h are more representative of the typical durations and intensities associated with team sport activity (e.g., soccer), half-marathon, marathons and road cycling stages. Importantly, it is also suggested that the upper limit of CHO intake could increase from 90 to 120 g/h (at least in trained participants), considering that both exogenous and whole-body rates of CHO oxidation can be increased with these higher ingestion rates [19,23,24].

Although our theoretical model to guide CHO ingestion also considers the whole-body CHO oxidation rates that are aligned to the maintenance of exercise intensity for a given duration, we readily acknowledge that the cited absolute CHO oxidation rates are reflective from investigations conducted on male athletes. Accordingly, the need to provide comparable data from female athletes is recognized as a major research priority. Furthermore, it is noteworthy that even with these higher rates of CHO ingestion, the sum of both endogenous and exogenous CHO availability is unlikely to provide all of the necessary substrate to sustain the required rates of energy production during exercise, especially as exercise duration increases beyond 2 h. As such, we consider that the high absolute rates of fat oxidation observed in marathoners [35], cyclists and ultraendurance athletes [165] (even at intensities associated with race pace) demonstrate that fat does not provide a negligible contribution to energy production, but rather, is indicative that fat provides an obligatory fuel source for elite endurance performance. When considered this way, training and nutritional strategies (e.g., train-low models aligned to CHO periodization and *fueling for the work required*) that optimize the capacity to oxidize both CHO *and* fat on race day would seem a major goal for the elite endurance athlete [135].

## Summary and Directions for Future Research

Despite >100 y of scientific research, the effects of CHO intake during exercise on substrate metabolism and exercise performance continue to be at the forefront of the nutrition-related performance questions posed by both the scientific and athletic community. In this regard, the apparent trend of higher-CHO intakes (i.e., >90 g/h) in elite endurance and ultraendurance athletes is in accordance with recent research developments, at least for ingestion rates ≤120 g/h. Nonetheless, considerable opportunities exist to advance our understanding of the optimal dose, frequency, ratio, and format of CHO ingestion that is ergogenic to male and female athletes, especially in consideration of the potential trade-off between optimizing CHO availability and any associated GI symptoms. In addition to such specific areas of investigation that are likely to inform “CHO personalization” strategies, there are also a number of bigger picture related themes that should be considered in future research.

**FIGURE 6:**
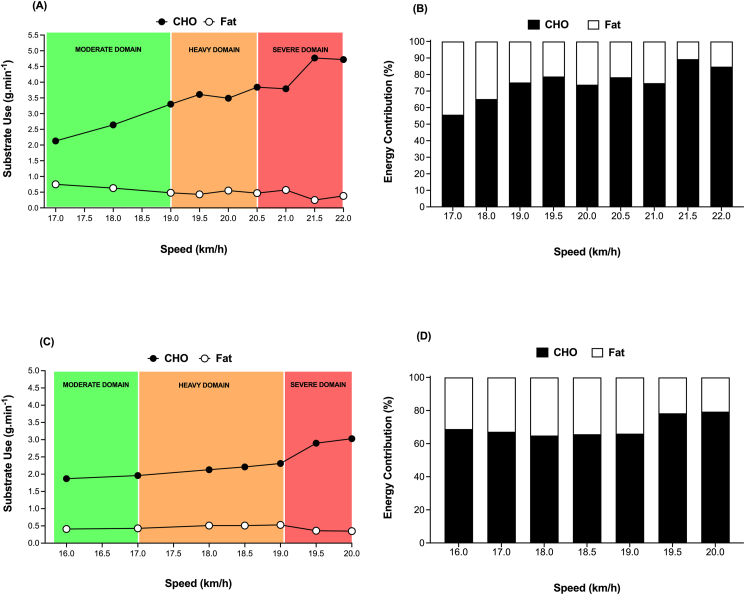
Absolute rate of CHO and fat utilization and contribution of CHO and fat to whole-body substrate metabolism in a tier 5 Ethiopian male (A and B, respectively) and female marathon runner (C and D, respectively). Data were collected during incremental exercise testing (3-min stages) on a motorized treadmill set at 1% incline (Pulsar H/p, Cosmos) in the corresponding author’s laboratory, where substrate utilization was assessed according to indirect calorimetry (Moxus Modular Metabolic System, AEI Technologies). Such data demonstrate the high capacity for fat oxidation in world-class athletes, even when running at the absolute speeds associated with marathon race pace. AEI, American Enterprise Institute; CHO, carbohydrate.

Indeed, the translational potential of original research will only ever be as good as the relevance of the training status of the research participants, the nutritional intervention and the validity of the exercise protocol to the situation of the intended end-user [183]. In the context of the elite endurance athlete and CHO feeding during exercise, it is noteworthy that recent research audits have demonstrated that <5% of research studies have been conducted on tier 4 (elite/international) and tier 5 athletes (world class) [184], as categorized according to the Participant Classification Framework [182]. As such, our current understanding of CHO feeding during exercise is largely based on studies (>80%) conducted on tier 1 (recreationally active) and tier 2 (trained/developmental) participants, of which the pattern of substrate utilization during exercise (especially the capacity for high absolute rates of fat oxidation) is likely to be very different than elite athletes (Figure 6). The requirement to study female participants (across all tiers of classification) is a clear research priority, especially when considering that <3% of studies have adopted female-only cohorts [80]. Accordingly, there is a definitive need to further characterize the habitual fueling practices and substrate metabolism of elite endurance and ultraendurance male and female athletes during both training and competition, the result of which should allow researchers to better design both laboratory and field-based studies [30] that evaluate the effects of CHO feeding strategies in conditions that are more applicable to real-world contexts. Furthermore, the mechanism(s) by which acute environmental stressors (e.g., heat and/or hyoxia) reduces exogenous CHO oxidation rates also remain to be determined, as does the potential for relevant acclimatization strategies to reverse such decrements.

In considering the difficulties associated with assessing outcome measures of competitive performance (i.e., reliable and valid testing protocols), such studies should also assess the effects of CHO availability on the underlying physiological determinants of performance that are of relevance to the specific sporting scenario e.g., economy, efficiency, durability, resilience, etc. Finally, when considering the necessity for high absolute rates of fat oxidation to sustain the required rates of energy production (even in the presence of high CHO availability), the interaction of training and nutritional strategies aligned to optimizing the capacity for both CHO (e.g., gut-training protocols) and fat oxidation (e.g., models of train-low and CHO periodization) is also considered an important area for future research. When taken together, it is clear that such a fundamental component of sports nutrition research and practice remains as exciting as ever, and it is hoped that this paper will stimulate collaborative efforts worldwide between scientists, athletes, coaches, and practitioners to further develop the personalization of CHO guidelines for athletes.

## Author contributions

The authors’ responsibilities were as follows – JPM: conceived the initial content and structure of the review with primary responsibility for final content; JPM, JTG, JMF, MAH: prepared figures; and all authors: contributed to the writing of this manuscript, read and approved the final manuscript and no formal funding was associated with the production of this review.

## Funding

The authors reported no funding received for this study.

## Conflict of interest

JPM reports a relationship with Science in Sport that includes: consulting or advisory and funding grants. JTG reports a relationship with Science in Sport that includes: consulting or advisory. JMF reports a relationship with Science in Sport that includes: employment. MAH reports a relationship with Science in Sport that includes: funding grants. JNP reports a relationship with Science in Sport that includes: consulting or advisory. JNP reports a relationship with ExoAnalytics that includes: equity or stocks. JTG reports a relationship with ZOE that includes: consulting or advisory. TP reports a relationship with Nduranz that includes: consulting or advisory. If there are other authors, they declare that they have no known competing financial interests or personal relationships that could have appeared to influence the work reported in this paper.
